# Physicians’ Perceptions and Demand Regarding Clinical and Secondary Use of Patient-Generated Health Data: Cross-Sectional Survey

**DOI:** 10.2196/86368

**Published:** 2026-06-04

**Authors:** Ye-Eun Park, Sang Sook Beck, Yura Lee

**Affiliations:** 1 Department of Information Medicine University of Ulsan College of Medicine Asan Medical Center Seoul Republic of Korea; 2 Asian Institute for Bioethics and Health Law General Complex B/D Graduate School of Public Health Yonsei University Health System Seoul Republic of Korea

**Keywords:** data management, data utilization, electronic health records, medical record linkage, patient access to records, patient-generated health data, patient-centered care, personal health records, telemedicine

## Abstract

**Background:**

Patient-generated health data (PGHD) are increasingly recognized as valuable for clinical care and secondary use; however, physicians’ perspectives remain heterogeneous and context-dependent.

**Objective:**

This study aimed to examine physicians’ perceptions, intentions, and concerns about the clinical and nonclinical use of PGHD and to compare responses between tertiary hospitals and other institutions.

**Methods:**

A cross-sectional survey was conducted involving 157 physicians (81 from a tertiary hospital and 76 from other health care institutions), supplemented by in-depth interviews. Between-group differences were assessed using chi-square tests and Wilcoxon rank-sum tests. Priority-ranking items were analyzed using weighted scores.

**Results:**

Overall, 64% (100/157) of the physicians reported prior use of PGHD, most commonly in handwritten or printed form, with no significant institutional differences; physiological measurements were the most frequent type of data. Among nonusers, 78.9% (45/57) reported that they had never received PGHD from patients, while poor system integration was identified as a major barrier to clinical use. Physicians cited clinical necessity and user-friendly integration as the strongest motivators. Perceived benefits focused on enriched decision-making, whereas key concerns included data accuracy, increased workload, and insufficient evidence. For nonclinical use, research and public health were viewed as most valuable. Across institutions, secure and transparent governance was prioritized. Differences between tertiary and nontertiary settings appeared to reflect physician demographics rather than divergent attitudes. Interviews further revealed that PGHD use was highly context dependent, with older adults’ limited digital literacy and discrepancies between patient- and caregiver-reported data identified as distinctive barriers in routine practice.

**Conclusions:**

Physicians recognize the potential of PGHD to support outpatient care, shared decision-making, and research, but express strong concerns about data reliability, workflow burden, and insufficient infrastructure. Differences between tertiary and nontertiary physicians reflect demographic and career stage imbalances, but both groups converge on the need for standardization, secure integration, and supportive governance frameworks. Broad adoption will require not only technical integration but also regulatory reform and trust-building strategies to advance biomedical research and health care delivery.

## Introduction

The health care paradigm is shifting from provider-centered care to patient-centered care. Although patient-centered care is not a new concept, it has gained renewed attention due to factors such as the rise of digital health and the increasing use of wearable devices [1,2]. Patient-generated health data (PGHD) has emerged as a key tool [3] that can allow the real-time collection of patient-reported symptoms and biometric data, tailored to individual needs [4]. This capability is particularly impactful for chronic disease management, where continuous monitoring of patients’ health status allows for timely interventions, ultimately reducing health care costs and improving clinical outcomes [5].

One key implication of this shift is the link between PGHD and reliable clinical information, such as electronic health records (EHRs). When integrated with EHRs, PGHD offers a more comprehensive view of a patient’s health status [6,7]. Although self-reported health and treatment histories are widely needed in clinical fields [8,9], challenges remain in terms of the effective integration and use of PGHD in health care institutions [6,10,11]. A critical factor in this integration is the attitude and decision-making of physicians [12,13].

Incorporating PGHD for meaningful patient participation requires the support and understanding of health care professionals, along with strong legal and systematic frameworks. However, previous studies have indicated that issues such as a lack of integration with EHRs, technostress, time pressure, and workflow-related challenges can exacerbate physician workload [6,11]. In addition, legal challenges and concerns regarding data reliability and quality remain barriers to clinical practice [14,15]. Although addressing and understanding these factors are essential for the successful integration of PGHD, research on physicians’ perceptions of PGHD, clinical applicability, and secondary uses (eg, research, commercial, and industrial purposes) remains limited [13,16]. A recent comprehensive review of health care professionals’ attitudes toward PGHD published in 2025 identified only 33 relevant studies conducted over the past decade, highlighting the limited empirical evidence in this area [17].

Therefore, we conducted a qualitative and quantitative analysis of physicians’ perceptions of the clinical use of PGHD through a web-based survey and in-depth interviews. The purpose of this study was to identify ways to improve data integration, suggest policy directions, and guide the safe and effective use of PGHD in clinical practice.

## Methods

### Study Design

This study adopted a mixed methods approach integrating a cross-sectional survey with in-depth interviews. The study followed an explanatory sequential mixed methods design, in which quantitative survey data were collected and analyzed first to identify general patterns in physician experiences and perceptions of PGHD. Subsequent qualitative interviews were informed by the survey findings and conducted to explore the underlying reasons, contextual factors, and practical interpretations of these quantitative results. Integration of quantitative and qualitative data occurred at the interpretation stage, and qualitative findings were used to contextualize and enrich the survey results rather than to independently generate or test new hypotheses.

### Survey Instrument Development

The questionnaire was designed to investigate physicians’ perspectives on the use of PGHD for various purposes, including consent processes, expectations, concerns, and suggestions for improvement. The development of the questionnaire involved direct discussion and consultation with a multidisciplinary panel of experts, including a medical regulatory and legal expert, medical informatics experts, a PGHD research specialist, and a health IT industry professional. The conceptual domains were informed by previous research conducted by the study team that examined patient perspectives on the use of PGHD; however, the physician survey instrument was independently developed, and no items were reused verbatim.

The finalized questionnaire comprised the following categories:

1. General Information (affiliated institution, medical specialty, years of clinical experience)
2. Experience with PGHD in clinical practice
3. Perspectives on using PGHD for clinical purposes
4. Perspectives on using PGHD for nonclinical purposes (eg, research or public health surveillance)
5. Perspectives on improving PGHD regulations

Response formats for each survey item employed various formats tailored to the specific information being sought. These formats included 5-point Likert scales (1=Not possible at all/Not helpful at all to 5=Always possible/Very helpful), yes/no responses, single-choice selections, multiple-choice selections with prioritization, and open-ended responses.

### Survey Respondents and Recruitment

A web survey was conducted from November 6 to 12, 2023, targeting physicians who currently use or have the potential to use PGHD. Recruitment was conducted using a structured approach during a predefined survey period. Study announcements describing the study purpose, eligibility criteria, and survey access link were distributed through official communication channels of national and specialty-specific associations and professional societies (eg, the Korean Association of Private Practitioners and the Korean Association of Geriatric Hospitals) and relevant societies (eg, the Korean Society of Medical Informatics and the Korean Society for Patient Safety).

Physicians accessed the survey via a web or mobile link or QR code and first received an electronic informed consent form. Only those who provided their consent were able to proceed with the questionnaire. Participation was voluntary and, upon completion of the survey, participants received a modest incentive (approximately US $8) as compensation for their time. Duplicate submissions were identified and removed during data cleaning, and responses from individuals who did not meet the eligibility criteria (eg, nonphysician affiliations) were excluded prior to analysis.

Participants were categorized into three distinct subgroups according to their respective work environments.

1. Primary and secondary health care facilities: (1) clinics with fewer than 30 beds and (2) hospitals with more than 30 but fewer than 100 beds
2. Tertiary and quaternary health care facilities: (1) general hospitals with more than 100 beds and (2) tertiary general hospitals
3. Nursing homes

Because this study was designed as an exploratory cross-sectional survey without a single primary hypothesis for formal hypothesis testing, a pragmatic sample size approach was adopted rather than a strict power-based calculation. Considering the planned descriptive analyses and multivariable exploratory analyses, our aim was to include at least 150 completed responses in the final analysis, which was considered sufficient to provide stable estimates of key survey items and to allow preliminary subgroup comparisons [18]. Assuming an analyzable response rate of approximately 75%, 200 health care professionals were approached to achieve the target final sample size.

During the survey period, 164 responses were collected. After excluding duplicate responses (n=4) and ineligible respondents (nonphysicians, n=3), the final analytical sample consisted of 157 participants. To minimize selection and response bias, the survey was administered anonymously using standardized procedures, and participants were recruited through multiple professional societies. For qualitative analysis, independent coding and reflexive discussions between researchers were used to minimize interpretive bias.

### Data Analysis

The survey responses were analyzed to identify general trends and exploratory differences between physicians working in tertiary hospitals and those working in other health care settings. Binary or categorical variables were analyzed using chi-square tests. Likert-scale items were treated as ordinal variables and analyzed using the Wilcoxon rank-sum test, a nonparametric method appropriate for ordered response data that does not assume normality. Continuous variables were also analyzed using the Wilcoxon rank-sum test. Statistical significance was defined as a 2-sided *P* value of <.05.

For priority ranking items, responses were weighted according to rank (3 points for first priority, 2 points for second priority, and one point for third priority), and the weighted scores were summed across respondents. All statistical analyses were performed using R (version 4.3.0; R Foundation for Statistical Computing). Subgroup analyses were conducted for exploratory purposes to identify descriptive patterns rather than estimate independent institutional effects; accordingly, analyses were not adjusted for demographic variables such as age, sex, or years of clinical experience.

Given the exploratory nature of the study, no formal adjustments for multiple tests were applied. The findings were interpreted on the basis of the direction of the effect, consistency across related items, and integration with qualitative insights rather than on statistical significance alone. For key single-choice outcomes, proportions are presented with 95% CIs calculated using the Wilson score method. CIs were not applied to descriptive multiple-response items. Missing data were minimal and were handled using complete-case analysis, with only respondents providing data for relevant variables included in each analysis, and no imputation was performed.

### In-Depth Interviews

After completion of the survey, in-depth interviews were conducted using a semistructured interview guide developed based on the survey questionnaire and key quantitative findings. The interviews were designed to explore why physicians perceived specific barriers, expectations, and concerns about the use of PGHD identified in the survey, rather than to compare perspectives across medical specialties.

Four individual interviews and one joint interview were conducted between August and November 2024. The joint interview involved 2 physicians of the same medical specialty working in different health care settings and was conducted to facilitate discussion between institutional contexts. Each interview lasted approximately 40-60 minutes and was conducted in person or via video conferencing, depending on participant availability and preference.

The interview participants were selected based on prior experience with PGHD in clinical practice, extensive clinical experience, nonparticipation in the survey (for triangulation), and diverse clinical backgrounds. The interviews were conducted in Korean by 2 members of the research team (YL and YEP), both trained in qualitative research and medical informatics. Neither interviewer had a prior personal supervisory relationship with the participants. All interviews were recorded with the consent of the participants and transcribed verbatim in Korean. The translated excerpts presented in the manuscript were translated into English during the preparation of the manuscript.

Qualitative data were analyzed using an inductive reflexive thematic analysis approach. Two researchers (YL and YEP) independently reviewed the transcripts and conducted an initial open coding to identify meaningful units related to experiences, perceived barriers, facilitators, and contextual factors associated with the use of PGHD. The codes were iteratively discussed and refined in team meetings, and higher-level themes were developed through consensus. Reflexive discussions were conducted throughout the analytic process to minimize interpretive bias and to ensure that the themes were grounded in the data. Given the exploratory and complementary role of the qualitative component, formal assessment of thematic saturation was not a primary analytic goal; interviews were conducted until sufficient explanatory depth was achieved to contextualize key quantitative findings.

### Ethical Considerations

This study was approved by the Institutional Review Board of Asan Medical Center, Korea (Institutional Review Board 2023-1363) and conducted in accordance with relevant ethical guidelines. Informed consent was obtained from all participants before their involvement, with assurances of anonymity and confidentiality. Participants were briefed on the study’s objectives and the intended use of the collected data. Participants received approximately US $8 in compensation for their participation.

## Results

### Cross-Sectional Survey

A total of 157 physicians participated in the survey, with 81 respondents (51.6%) from a tertiary general hospital and 76 (48.4%) from other health care institutions. The 2 groups differed significantly in demographic makeup (Table 1). Additionally, we identified participants’ specialties, with the total sample consisting of 29 respondents from internal medicine, 17 from neurology, 16 from family medicine, 12 from emergency medicine, 15 from surgery, and 68 from other departments.

**Table 1:** Characteristics of respondents by type of health care institution.

Variables			Tertiary general hospital (81/157, 51.6%), n (%)		Other health care institutions^a^ (76/157, 48.4%), n (%)		Total (N=157), n (%)		*P* value
**Sex**									.01
	Male	32 (39.5)		45 (59.2)		77 (49)			
	Female	49 (60.5)		31 (40.8)		80 (51)			
**Age**									<.01
	20s	25 (30.9)		5 (6.6)		30 (19.1)			
	30s	39 (48.1)		24 (31.6)		63 (40.1)			
	40s	16 (19.8)		41 (53.9)		57 (36.3)			
	≥50 years	1 (1.2)		6 (7.9)		7 (4.5)			
**Length of work experience (years)**									.01
	<2	11 (13.6)		7 (9.2)		18 (11.5)			
	2-4	27 (33.3)		11 (14.5)		38 (24.2)			
	5-9	20 (24.7)		19 (25)		39 (24.8)			
	≥10	23 (28.4)		39 (51.3)		62 (39.5)			
**Type of health care institution**									
	General hospital	—^b^		40 (52.6)		—		—	
	Clinic	—		24 (31.6)		—		—	
	Nursing home	—		12 (15.8)		—		—	

^a^Other health care institutions include primary and secondary health care facilities and nursing homes.

^b^Not applicable.

The demographic data collected were analyzed based on the length of work experience, categorizing the respondents into 2 groups: those with less than 5 years of experience (56/157, 35.7%) and those with 5 years or more (101/157, 64.3%). A comparison between the groups revealed significant differences in sex (*P*=.03), age range (*P*<.01), and type of affiliation (*P*<.01).

### Single-Choice and Multiple-Response Analysis

Overall, 64% (100/157) of the surveyed physicians reported having used PGHD in their clinical practice (Table 2). This proportion was almost identical between tertiary hospital doctors and those in other settings (52/81, 64.2% vs 48/76, 63.2%; *P*=.89), with no significant difference by institution type. Among the 100 physicians who had used PGHD, the most common format in which patients provided data was a handwritten paper record, reported by 65% (65/100). Physicians who had not used PGHD (57/157, 36.3%) were asked for the main reason. The vast majority (45/57, 78.9% overall) indicated that they had simply never received self-recorded health data from any patient. A few physicians (5/57, 8.8% overall) reported that patients had offered such data, but it was not helpful for treatment, and a similar fraction (5/57, 8.8%) said they had seen patient data but did not have enough time to review it during visits. There was a noticeable but not significant difference in these reasons between institution types (*P*=.06).

**Table 2:** Physician experiences with patient-generated health data (PGHD) by type of health care institution.

Questions			Tertiary general hospital (81/157, 51.6%)		Other hospitals^a^ (76/157, 48.4%)		*P* value			Total (N=157)	
**B1. Have you used health information recorded by patients during your clinical practice (other than verbal symptom descriptions or records from other medical institutions)?**								.89			
	Yes^b^, n (%, 95% CI)	52 (64.2, 53.3-73.8)		48 (63.2, 51.9-73.1)					100 (64, 55.9-70.8)		
	No, n (%)	29 (36)		28 (37)					57 (36)		
**B1a. If you answered yes, in what format were the records provided by the patient? (Select all that apply) (n=100)^c^, n (%)**											
	Handwritten records on paper	31 (59.6)		34 (70.8)		.51			65 (65)		
	Records printed on paper	17 (32.7)		21 (43.8)		.43			38 (38)		
	Records of webpages or apps connected to electronic health records (EHR)	18 (34.6)		13 (27.1)		.55			31 (31)		
	Patient’s smartphone/tablet/laptop	12 (23.1)		14 (29.2)		.70			26 (26)		
	Other	0		1 (2.1)		.97			1 (1)		
**B1b. If you answered yes, what information did the patient provide in the records? (Select all that apply) (n=100), n (%)**											
	Measurements include body temperature, blood pressure, or blood sugar	34 (65.4)		33 (68.8)		.98			67 (67)		
	Continuous records of symptoms (eg, pain, nausea, defecation, or urination)	27 (51.9)		26 (54.2)		>.99			53 (53)		
	Continuous records of health care/self-care (eg, medications, wound care, exercise, or diet)	23 (44.2)		18 (37.5)		.62			41 (41)		
	Physical measurements such as height, weight, or BMI	19 (36.5)		20 (41.7)		.82			39 (39)		
	Vital sign information includes oxygen saturation, pulse rate, or electrocardiogram	13 (25)		10 (20.8)		.78			23 (23)		
	Other	3 (5.8)		1 (2.1)		.66			4 (4)		
**B1c. If you answered** **no** **, what is the reason? (n=57), n (%)**								.06			
	I have never received self-records from patients	27 (93.1)		18 (64.3)					45 (78.9)		
	I have received them, but they were not helpful for treatment	1 (3.4)		4 (14.3)					5 (8.8)		
	I have seen them, but time was insufficient to review them	1 (3.4)		4 (14.3)					5 (8.8)		
	Other	0		2 (7.1)					2 (3.5)		
**B2. Does your health care organization offer or operate patient health records (smartphone app/webpage)?**								<.01			
	Yes, n (%, 95% CI)	19 (23.5, 15.6-33.8)		9 (11.8, 6.4-21.0))					28 (17.8, 12.6-24.6)		
	No, n (%)	21 (25.9)		53 (69.7)					74 (47.1)		
	I do not know, n (%)	41 (50.6)		14 (18.4)					55 (35)		

^a^Other health care institutions include primary and secondary health care facilities and nursing homes.

^b^95% CIs are provided only for key single-choice outcomes.

^c^For multiple-response items (B1a and B1b), the percentages are based on the respondents who reported prior use of PGHD, and the CIs are not shown to maintain clarity.

The most frequently reported PGHD were home-recorded physiological measurements, such as body temperature, blood pressure, or blood sugar (67/100, 67%). Symptom diaries (eg, logs of pain, nausea, or other symptoms) were the next most common (53/100, 53%), followed by records of self-care activities such as medication adherence, wound care, exercise, or diet (41/100, 41%). About 39% (39/100) mentioned physical metrics such as body weight or BMI. Only a small share (23/100, 23%) had encountered continuous vital sign data (eg, oxygen saturation or heart rate trends) from patients. Notably, there were no significant differences by hospital type in the kinds of PGHD provided by patients (all *P*>.5).

Regarding the institution-derived patient health record (PHR) system (eg, a smartphone app or a web portal for patients), there was a significant disparity: Tertiary hospital physicians were more likely to report that such a system existed (19/81, 23.5% vs 9/76, 11.8% in other institutions), and far fewer of them stated outright that no patient portal was available (21/81, 25.9% vs 53/76, 69.7%; *P*<.01). However, awareness of these systems differed: Over half of tertiary hospital respondents (41/81, 50.6%) said they did not know whether their organization had a PHR app, whereas only 18.4% (14/76) of physicians in other settings were unsure.

In addition, physicians in both groups identified similar barriers to incorporating PGHD into current practice. In the category of survey exploring perspectives on the use of PGHD in clinical practice, no statistically significant differences were observed between respondents from tertiary general hospitals and other hospitals in all items analyzed (*P*>.05) (Table 3).

Despite the lack of significant differences, both groups showed similar trends in their responses. When asked about the main barriers to the integration of PGHD collected in nonclinical settings with the EHR, the most common reason cited in both groups was system-based limitations (C1a). This represented 27.2% (22/81) of respondents from tertiary hospitals, 35.5% (27/76) from other hospitals, and 31.2% (49/157) overall. The most significant motivation for enabling PGHD–EHR integration was identified as the need/demand for PGHD utilization (C1b). This was reported by 48.1% (39/81) of respondents from tertiary hospitals, 40.8% (31/76) from other hospitals, and 44.6% (70/157) overall.

Furthermore, the most frequently cited reason for the benefits of PGHD utilization was its high value for providing meaningful insights into patients’ health conditions (C2b). This was reported by 43.1% (28/65) of tertiary hospital respondents, 25.5% (14/55) of other hospital respondents, and 35% (42/120) overall. By contrast, the most commonly cited drawback of PGHD utilization was its unintended negative effects, such as prolonged consultations or inaccurate decision-making (C2a). This accounted for 38.3% (31/81) of responses from tertiary hospitals, 43.4% (33/76) from other hospitals, and 40.8% (64/157) overall.

**Table 3:** Distribution of responses on intent to use patient-generated health data (PGHD) for practice by type of health care institution.

Questions			Tertiary General Hospital (81/157, 51.6%), n (%)		Other hospitals^a^ (76/157, 48.4%), n (%)		*P* value			Total (N=157), n (%)	
**C1a. In the current health care setting, what is the main reason it is difficult to use PGHD (produced and recorded in nonclinical settings) in combination with medical records?**								.21			
	System-based: Not available for use during treatment (lack of integration of PGHD-EHR^b^ or provision of summary/visualized dashboard)	22 (27.2)		27 (35.5)					49 (31.2)		
	Time/cost: Absence of relevant compensation (fees) and lack of treatment time	18 (22.2)		20 (26.3)					38 (24.2)		
	Rationale/accountability: Unclear accountability for PGHD-based decisions or lack of guidance or guidelines to make judgments based on PGHD	20 (24.7)		16 (21.1)					36 (22.9)		
	Current state of utilization: Little or no PGHD is collected or provided	13 (16)		7 (9.2)					20 (12.7)		
	Absence of need/demand: No clinically valuable information is available	4 (4.9)		6 (7.9)					10 (6.4)		
	Other	4 (4.9)		0 (0)					4 (2.5)		
**C1b. In the current health care setting, what is the biggest motivation to use PGHD in combination with medical records?**								.58			
	Demand: High necessity/high willingness (of physicians and/or patients) to use	39 (48.1)		31 (40.8)					70 (44.6)		
	System-infrastructure: PGHD that is well integrated into the information system or dashboards that provide easy-to-understand summaries	15 (18.5)		23 (30.3)					38 (24.2)		
	Time/cost: Reward (fees) or there is sufficient time	9 (11.1)		10 (13.2)					19 (12.1)		
	Current utilization environment: Many patients are already collecting PGHD	11 (13.6)		7 (9.2)					18 (11.5)		
	Rationale/accountability: The use of PGHD was recommended in guidelines set by academic societies, treatment-related organizations, or colleagues	6 (7.4)		4 (5.3)					10 (6.4)		
	Other	1 (1.2)		1 (1.3)					2 (1.3)		
**C2a. (This question asks for your opinion regardless of your response to the previous question.) If it is not helpful, what is the main reason? (N=157)**								.82			
	Unintended negative effects: Incorrect decisions were made, or unnecessary conversations with patients were prolonged	31 (38.3)		33 (43.4)					64 (40.8)		
	Lack of means: Information was overwhelming or unavailable during treatment	27 (33.3)		25 (32.9)					52 (33.1)		
	Rationale: Lack of evidence (research results) to make a judgment based on PGHD	15 (18.5)		9 (11.8)					24 (15.3)		
	Lack of value: No clinically valuable information	6 (7.4)		7 (9.2)					13 (8.3)		
	Other	2 (2.5)		2 (2.6)					4 (2.5)		
**C2b. If it was helpful, what was the main reason? (n=120)^c^**								.22			
	High value: PGHD is inherently valuable for understanding patient health conditions and making treatment decisions	28 (43.1)		14 (25.5)					42 (35)		
	Indirect positive effects: Improved trust relationships with patients and improved adherence to treatment	16 (24.6)		20 (36.4)					36 (30)		
	Synergies: There are more positive effects or efficiencies when the EHR and PGHD information is integrated	16 (24.6)		15 (27.3)					31 (25.8)		
	There is evidence (research results) to make judgments based on PGHD	5 (7.7)		6 (10.9)					11 (9.2)		
	Other	0 (0)		0 (0)					0 (0)		

^a^Other health care institutions include primary and secondary health care facilities and nursing homes.

^b^EHR: electronic health record.

^c^For C2b, the percentages are calculated based on respondents who reported that PGHD was helpful (tertiary hospitals: n=65; other hospitals: n=55; total: n=120).

**Figure 1:**
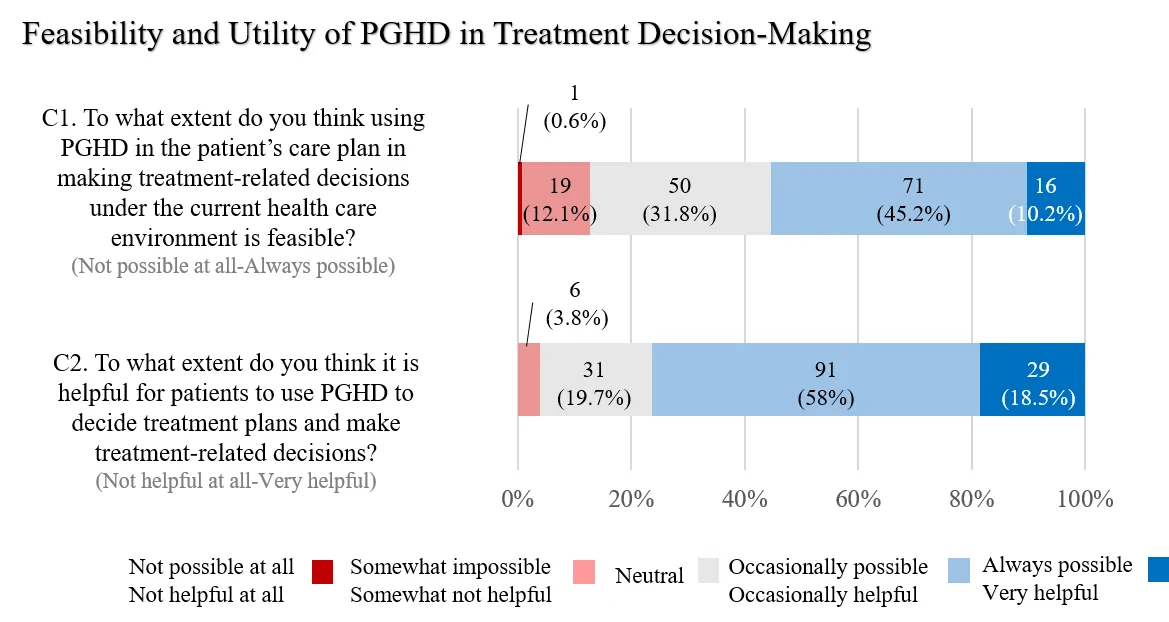
Distribution of survey responses to C1 and C2 questions (N=157). PGHD: patient-generated health data.

We assessed the feasibility of using PGHD integrated with EHR for treatment planning and clinical decision-making under current health care conditions. As shown in Figure 1, “Occasionally possible” was the response selected most frequently (71/157, 45.2%), whereas 55.4% (87/157) of respondents provided positive responses (C1). No statistically significant difference was observed between tertiary hospitals and other hospitals (*P*=.61) based on the Wilcoxon rank-sum test.

Regarding the perceived benefits of the use of PGHD, the most frequently selected response was “Occasionally helpful” (91/157, 58%). Overall, 76.4% (120/157) of respondents expressed positive responses, including “Occasionally helpful” and “Highly helpful” responses (C2). Similarly, no statistically significant difference was identified between tertiary hospitals and other hospitals (*P*=.08), as determined by the Wilcoxon rank-sum test.

Looking beyond direct clinical use, there was a clear preference for using PGHD for public good and knowledge generation rather than commercial purposes. The highest-ranked secondary use was academic, that is, using PGHD for research, education, or developing clinical guidelines, with a total score of 329. The second priority was public health and national statistics, with a score of 270, highlighting that many physicians see value in aggregating PGHD to inform population health monitoring or policy. By comparison, industry-related uses (eg, technology development, drug discovery, or algorithm training) received moderate support, with a score of 174. Commercial purposes, such as recommending personalized health services and providing personalized advertisements, were less favored, with a score of 164. The least prioritized use of PGHD was the trade and sale of health information data, with a score of 5.

To enable these uses of PGHD, respondents highlighted several areas for improvement in governance and infrastructure (Table 4, Question D2). The top priority was enhanced data security and personal information protection, with a weighted score of 263. This aligns with the expectation that if PGHD is to be widely used, especially outside one-on-one care, robust safeguards must be in place to protect patient privacy. The second priority was ensuring transparency, with a score of 219, followed by improvement of the consent system, with a score of 205. For regulatory or policy changes to facilitate the use of PGHD (Table 4, Question E1), 2 clear frontrunners emerged: data standardization, with a score of 204, and increased security obligations for data management companies, vendors, and government support policies, with a score of 196.

**Table 4:** Expectations, concerns, and improvement requirements for the use of patient-generated health data (PGHD): priority response items for all respondents (N=157).

Questions and rank/response items		Total weighted score^a^
**C3. What are your main concerns about using PGHD for medical treatment?**		
	1. Issues related to data accuracy.	322
	2. Increased clinical workload.	164
	3. Problems with confidence in a clinical setting and a lack of evidence for data analysis results.	164
	4. Lack of guidelines on recording PGHD by integrating with medical records.	139
	5. Accountability issues for PGHD-based decisions.	73
	6. Issues related to the protection and security of personal information.	70
**D1. From a physician’s perspective, which of the following purposes is the most important and necessary when using PGHD for purposes other than medical treatment?**		
	1. Academic purposes, such as research, education, and practice guideline development.	329
	2. Public interest purposes, such as public health and national statistics.	270
	3. Industrial purposes include technology development, drug discovery, and algorithm development.	174
	4. Commercial purposes, such as recommending personalized health services and providing personalized advertisements.	164
	5. Trade and sale of health information data.	5
**D2. What are the most pressing areas of improvement to use PGHD for nonclinical purposes?**		
	1. Enhanced security/personal information protection.	263
	2. Securing transparency.	219
	3. Improvement of the consent system.	205
	4. Evaluation/compensation.	157
	5. Preparation of verification measures for user reliability.	98
**E1. Related regulations need to be improved for the use of PGHD for treatment or research. Which of the following should be prioritized for revision?**		
	1. Data standardization.	204
	2. Increased security obligations for data management companies/vendors and government support policies.	196
	3. Clarification of the right to information transfer in the Medical Service Act.	138
	4. Preparation of an article on exceptions for not sending information despite a patient’s request.	119
	5. Incentives for related maintenance costs of the system.	82
	6. Support for follow-up response policies in the event of an incident, such as a data breach.	80
	7. Regulations for securing interoperable systems.	65
	8. Technologies for data pseudonymization/deidentification.	58

^a^Total weighted score was calculated by assigning 3 points to first-priority selections, 2 points to second-priority selections, and 1 point to third-priority selections, and summing the points across all respondents.

### In-Depth Interviews

In-depth interviews included a total of 6 participants, consisting of 3 female and 3 male physicians. All participants had over 10 years of clinical experience and were affiliated with either tertiary referral hospitals (n=4) or clinics (n=2). Their specialties were diverse, including emergency medicine (n=1), breast surgery (n=1), critical care/trauma surgery (n=1), geriatrics (n=1), and pediatrics (n=2). The 2 pediatricians participated in a joint interview, whereas the remaining 4 physicians underwent individual interviews (Table 5).

**Table 5:** In-depth interviewee characteristics.

ID	Affiliation	Specialty	Sex
P1	Clinic (less than 30 beds)	Emergency medicine	Female
P2	Tertiary referral hospital	Surgical oncologist (breast cancer)	Female
P3	Tertiary referral hospital	Critical care/trauma	Male
P4	Tertiary referral hospital	Geriatrics	Male
P5	Clinic (less than 30 beds)	Pediatrics	Male
P6	Tertiary referral hospital	Pediatrics	Female

In qualitative interviews, all participating physicians reported prior experience using PGHD in clinical practice, primarily in the context of follow-up care and chronic disease management. PGHD was commonly integrated into outpatient workflows through patient-maintained records, such as symptom diaries, home-measured physiological data, and self-care behavior logs. Physicians noted that the quality and availability of PGHD were often influenced by patient characteristics, including personality traits and, in some cases, discrepancies between patient and caregiver-reported data, particularly in geriatric populations. In tertiary care settings, PGHD also included externally generated data, such as laboratory results from other institutions or patient-submitted images.

Physicians consistently identified several barriers to the use of PGHD. The challenges cited most frequently included limited consultation time and workflow constraints, which restricted the ability to review patient-provided data in detail. Concerns about data reliability and device accuracy were also prominent, as were issues related to excessive data volume and a lack of quality control. Furthermore, factors related to the patient, particularly limited digital literacy among older adults, were perceived as significant obstacles to the effective use of PGHD.

Despite these challenges, participants emphasized multiple facilitators that support the use of PGHD. Clinical need was described as the main driver, particularly for conditions that require continuous monitoring beyond episodic clinical encounters. Physicians highlighted the role of PGHD in enabling remote management, enhancing patient safety, and supporting shared decision-making. The availability of evidence-based guidelines and accountability requirements further reinforced the use of PGHD. Importantly, participants underscored the high value of PGHD as a source of real-world data that complements traditional EHR data by providing longitudinal insights. Additional benefits included improved patient engagement and motivation, as well as synergistic integration with existing clinical information systems.

Physicians expressed a range of concerns about the clinical use of PGHD, with data accuracy identified as the most critical issue. Participants emphasized that variability in the patient context and measurement conditions could compromise the reliability of PGHD. The increased clinical workload associated with the review and interpretation of patient-generated data was also a major concern, along with the potential legal and ethical liabilities when clinical decisions are based on uncertain data sources.

Beyond direct clinical care, physicians identified academic research as the most important secondary use of PGHD, followed by contributions to the public interest, such as informing health policy and population-level statistics. Industry applications were considered of relatively lower priority. Participants highlighted the potential of PGHD to generate real-world evidence and to supplement existing data sources, particularly in underrepresented populations, such as pediatric patients.

Regarding nonclinical use, physicians emphasized the need for improvements in data security, transparency, and consent processes. Ensuring robust data protection and clear communication about how data are used was considered essential for building trust. Participants also stressed the importance of establishing mechanisms to verify the trustworthiness of the organizations that handle PGHD.

In terms of regulatory priorities, participants highlighted the importance of improving patient data portability and strengthening requirements for data security and standardization to support interoperability. Several physicians also emphasized the need to simplify consent procedures, noting that available processes are often too complex and can hinder the practical use of PGHD in both clinical and research settings.

### Integrated Findings

The qualitative findings largely converged with the survey results and provided additional context for the main quantitative patterns. Table 6 presents a joint display integrating the quantitative and qualitative findings.

**Table 6:** Joint display integrating quantitative and qualitative findings on physicians’ perceptions of patient-generated health data (PGHD).

Domain	Quantitative findings	Qualitative findings	Integrated interpretation
Current use and format of PGHD	Overall, 64% of physicians reported prior use of PGHD, with no significant institutional difference (64.2% in tertiary hospitals vs 63.2% in other institutions). Handwritten paper records were the most common format, and home-recorded physiological measurements were the most frequently encountered type of PGHD.	Interview participants reported using PGHD mainly in follow-up care and chronic disease management through symptom diaries, home-measured physiological data, and self-care behavior logs. In tertiary settings, PGHD also included externally generated data such as laboratory results from other institutions or patient-submitted images.	PGHD is already used in routine practice, but mostly through clinician-mediated and low-integration workflows rather than seamless digital systems.
Barriers to clinical use	The most commonly reported barrier to using PGHD with medical records was system-based limitation, including the lack of PGHD-EHR^a^ integration or the absence of summary/dashboard tools. Other frequently reported concerns included unintended negative effects, information overload, lack of evidence, data accuracy, and increased clinical workload.	Interviewees emphasized limited consultation time, workflow constraints, data overload, device accuracy concerns, lack of quality control, and patient-level barriers such as older adults’ limited digital literacy and discrepancies between patient- and caregiver-reported data.	Survey-identified implementation barriers were reinforced by interview findings showing that PGHD use is constrained not only by technical limitations but also by workflow- and patient-level factors in routine care.
Facilitators and perceived clinical value	The strongest motivation for PGHD use was clinical necessity or willingness to use PGHD, followed by user-friendly system integration. Physicians most often viewed PGHD as helpful because it provided valuable information for understanding patient health conditions and informing treatment decisions.	Interview participants likewise described clinical need as the main driver, especially for conditions requiring continuous monitoring beyond episodic encounters. They also highlighted PGHD’s role in remote management, patient safety, shared decision-making, patient engagement, and synergistic use with existing clinical information systems.	PGHD is perceived as most useful when there is a clear clinical purpose and when the information can be meaningfully incorporated into care workflows.
Secondary use of PGHD	For nonclinical purposes, academic use ranked highest, followed by public-interest use, such as public health and national statistics. Industry-related use received lower support, and trade or sale of health data was the least acceptable.	Interviewees similarly prioritized research and public-interest uses over industry applications and described PGHD as a source of real-world longitudinal data, particularly useful for underrepresented groups such as pediatric patients.	Quantitative and qualitative findings converged in showing that physicians support secondary use of PGHD primarily when it serves knowledge generation or public benefit rather than commercial exploitation.
Governance and regulatory priorities	For nonclinical use, the top priorities were stronger security, greater transparency, and improved consent systems. For regulatory improvement, the highest priorities were data standardization and stronger security obligations for organizations handling PGHD.	Interviewees stressed the need for robust data protection, transparent communication about data use, trust in the organizations handling PGHD, improved portability and interoperability, and simpler consent procedures for practical clinical and research use.	Broader adoption and secondary use of PGHD depend not only on technical infrastructure but also on trustworthy governance frameworks that support security, transparency, interoperability, and practical consent processes.

^a^EHR: electronic health record.

## Discussion

### Principal Results

This study explored the perceptions of physicians about the clinical and nonclinical use of PGHD. Our results suggest that although PGHD has the potential to play an important role in the health data ecosystem, its adoption is hindered by practical barriers. Physicians acknowledged the utility of PGHD for remote patient management and treatment planning but raised concerns about data reliability, excessive data volume, system integration challenges, and time and cost burdens. For nonclinical purposes, PGHD was perceived as more valuable for academic research and, to a lesser extent, for public-interest applications, such as policymaking. To support these uses, physicians emphasized the need for greater security, standardized data management, and clear regulatory frameworks.

Although the majority (100/157, 64%) of surveyed physicians reported having used PGHD in their clinical practice, the most common format was handwritten paper records (65/100, 65%). There was a significant disparity in the reported availability of institutional patient portals, with physicians in other institutions much more likely to report that no such system was available (53/76, 69.7% vs 21/81, 25.9%; *P*<.01). Nevertheless, relatively few physicians (31/100, 31%) had received PGHD through digital means, which was similar in both the tertiary and nontertiary groups (no significant differences, *P*>.4 for all). Moreover, awareness of these systems differed; over half of the tertiary hospital respondents (41/81, 50.6%) said they did not know whether their organization had a PHR app, whereas only 18.4% (14/76) of physicians in other settings were unsure. This pattern suggests that tertiary centers are more likely to have some PGHD infrastructure, but many staff may not be aware of it, implying that a significant proportion of health care professionals lack awareness of PHR utilization. This suggests, even before the data quality issues discussed later, that the amount of information available to physicians in digital form is relatively small, and physicians’ motivation to use PHR is limited.

Physicians in both groups noted system-related limitations, specifically, the lack of integration of patient data into the EHR or the absence of convenient tools (eg, summary dashboards) to review PGHD during clinical care. In contrast, relatively few respondents felt that the lack of PGHD was the main issue, only 12.7% (20/157) said the current lack of PGHD availability was the top barrier, and merely 6.4% (10/157) believed there was no need for or no valuable information in PGHD, with no significant difference by hospital type (*P*=.21). This consensus highlights that technical integration and resource constraints are seen as key hurdles to using PGHD in practice, rather than a lack of data or interest. The US Office of the National Coordinator for Health IT has outlined a modular architecture for PGHD integration, recommending the use of intermediary platforms (eg, data brokers or gateways) to facilitate data flow [19]. Additionally, the US Office of the National Coordinator for Health IT emphasizes the incorporation of clinical decision support tools to enable efficient triage and interpretation of patient-generated data within clinical workflows [19]. When considering what would most motivate or enable greater PGHD use, a clear clinical need or willingness for PGHD use emerged: respondents said that a high perceived need for PGHD (and strong willingness to use it) would be the biggest driver for adoption. Far fewer respondents prioritized financial or time incentives. It is worth mentioning, however, that physicians from smaller hospitals showed a trend toward valuing system improvements slightly more (23/76, 30.3% chose “well-integrated system” as the top motivator vs 15/81, 18.5% of those in tertiary hospitals), whereas tertiary hospital physicians were somewhat more likely to emphasize intrinsic need (39/81, 48.1% vs 31/76, 40.8% in others). Overall, however, both groups ranked clinical need the highest, followed by better systems, echoing a shared view that demand and infrastructure are pivotal for PGHD implementation.

Regarding the reasons why incorporating PGHD might not be helpful, the predominant concern (64/157, 40.8%) was the risk of unintended negative consequences. This included scenarios such as making incorrect clinical decisions based on inaccurate patient data or wasting time in protracted discussions about patient-collected information that would not benefit care. This is consistent with the C3 priority scores, in which physicians’ top concerns about using PGHD in practice were clearly centered on data quality. The next major reason was a lack of practical means to use the data. In the same vein as the second priority (ie, workflow implications), it might be overwhelming or not accessible in the right format at the point of care. In essence, without better tools or workflows, PGHD could be more of a hindrance than a help [11]. Notably, data privacy and security were the lowest ranked of the provided concerns, though the top priority was enhancing data security and personal information protection (Question D2; weighted score 263). This suggests that, in the context of day-to-day practice, clinicians are currently more focused on practical implementation challenges (accuracy, workload, and guidelines) than on legal and privacy ramifications. Given these practical constraints, health systems must establish formalized data quality assurance protocols and incorporate PGHD through clinically embedded infrastructures. The Veterans Health Administration, for instance, has adopted standardized operational procedures to support consistent data acquisition and interpretation across settings, whereas recent informatics frameworks advocate modular integration architectures and decision support functionalities aligned with existing clinical workflows [20,21].

Meanwhile, when asked why using PGHD would be helpful, physicians most frequently cited the direct informational value of PGHD. More than one-third (42/120, 35%) indicated that PGHD provides important insights into a patient’s health status and can inform better clinical decision-making. The highest-ranked secondary use (Question D1) was academics, that is, the use of PGHD for research, education, or developing clinical guidelines, which suggests that PGHD is expected to contribute to personalized care. Many also recognized indirect benefits: 30% (36/120) noted that engaging with PGHD can improve the physician–patient relationship, build trust, and improve patient engagement or adherence to treatment. Additionally, 25.8% (31/120) highlighted efficiency or synergy gains when PGHD is integrated with clinical systems. This refers to scenarios in which having patient data seamlessly integrated into EHRs could enhance monitoring or enable more personalized, timely interventions, thereby improving care processes. However, seamless care requires interoperability between data and systems. This is consistent with the findings of the E1 survey, which emphasized the importance of data standards. Physicians recognize the need for common standards and interoperability so that PGHD can be seamlessly integrated into medical records across different platforms and institutions. The second priority was strengthening data management policies, specifically by increasing security obligations for companies handling health data and providing governmental support. This echoes the earlier emphasis on security and indicates a desire for clearer regulations holding vendors accountable for protecting PGHD. This aligns with earlier findings: tackling technical integration (standards) and trust/safety issues (security and consent) is seen as foundational for successfully implementing PGHD at the system level.

Our qualitative interviews confirmed that PGHD is already used in clinical practice, albeit in uneven ways in all specialties. For example, oncologists described structured approaches using standardized forms for drainage or side effects, whereas primary care physicians noted that PGHD often depends on the personality and self-motivation of the patient. Geriatricians highlighted discrepancies between caregiver- and patient-reported data, especially in populations with dementia, whereas pediatricians in tertiary settings reported reliance on externally provided laboratory results when children could not attend in person. These findings echo previous survey and review studies showing that physicians are broadly supportive of PGHD but remain cautious due to variability in data quality and completeness [18,22,23].

The types of PGHD reported in our study, that is, symptom diaries, physiological measurements, and self-care logs, are consistent with the existing oncology and chronic disease management literature. Basch et al [24] found that the systematic collection of patient-reported outcomes improved quality of life and even survival in cancer patients, underscoring the clinical value of self-reported longitudinal data. However, many participants in our study emphasized the burden of interpreting raw PGHD without visualization or contextualization, supporting calls for improved integration with EHRs and decision-support systems [25].

Physicians also recognized that PGHD can strengthen patient engagement, although unevenly. Only patients who are organized, digitally educated, or supported by caregivers are likely to generate actionable PGHD. This reflects previous findings that older adults or patients with cognitive impairment require caregiver mediation to produce reliable data, whereas younger, digitally adept patients contribute more consistently [26].

Regarding nonclinical use, physicians in our study emphasized academic and public-interest purposes over industrial applications. Pediatricians noted that PGHD could fill the gaps in pediatric statistics, aligning with larger initiatives such as the European Health Data Space, which positions PGHD as a complement to traditional clinical data for research and public health [17,26]. However, barriers remain: Physicians ranked security, transparency, and consent frameworks as the most urgent priorities for reform, echoing concerns documented in systematic reviews of PGHD governance [27]. They also stressed the importance of verifying the trustworthiness of the entities handling PGHD, whether governmental, institutional, or commercial, underscoring the need for governance models that build both technical and ethical trust.

Finally, physicians prioritized data portability, stronger security obligations, and standardization as key areas for regulatory reform. These priorities mirror global policy trends, including interoperability and anti-information-blocking provisions in the US 21st Century Cures Act [28] and the General Data Protection Regulation’s emphasis on patient data portability in Europe [29]. Some participants also highlighted the burden of current consent requirements, noting that repeated signatures and stringent legal reviews create barriers to PGHD use in research. Similar challenges have been observed in studies of electronic and dynamic consent models, which aim to balance regulatory rigor with practical usability [30].

### Limitations

This study has several limitations that should be acknowledged. First, the number of respondents to the cross-sectional survey might not have been sufficient to fully represent the perceptions of physicians about PGHD in all specialties and clinical settings. Previous studies aimed at patients and caregivers have demonstrated that PGHD perceptions vary substantially depending on the type of disease and patient characteristics [31]. However, when designing our physician survey, we were unable to incorporate this level of diversity. To mitigate this limitation, we conducted in-depth interviews with physicians from a range of specialties and health care institutions, adding context and nuance to the survey findings.

Second, there were notable demographic imbalances between physicians in tertiary general hospitals and those in smaller institutions. Specifically, respondents from tertiary hospitals were younger and more likely to be trainees or early-career clinicians, whereas respondents from other hospitals tended to have more years of clinical experience. This imbalance likely reflects the real-world distribution of career stages between institution types; however, it may have introduced bias when comparing perceptions of PGHD between hospital groups. For example, age and career stage could influence digital literacy, openness to new technologies, or workload burden, which in turn might have affected the subgroup differences observed in our analyses. Consequently, comparisons between institution types should be interpreted as exploratory and descriptive, rather than as estimates of independent institutional effects.

Third, the cross-sectional nature of this study limited our ability to make causal inferences. Physicians’ perceptions and intentions regarding PGHD can evolve as institutional infrastructure, reimbursement policies, and national regulations develop. Longitudinal studies would be required to capture how attitudes change over time in response to these contextual factors.

Fourth, although our study combined quantitative and qualitative methods, self-selection bias may have influenced those who participated in both the survey and the interviews. Physicians with more interest in digital health may have been more inclined to respond, potentially overestimating the level of acceptance of PGHD among clinicians in general.

Finally, although our analysis highlighted significant differences between tertiary and nontertiary hospital physicians, we did not stratify the findings by specialty in a systematic way. Specialty-specific factors (eg, oncology vs psychiatry) likely shape the information needs of physicians and their perceptions of the value of PGHD. Future studies with larger and more diverse physician samples should examine specialty-level differences more rigorously.

Taken together, these limitations suggest that our findings should be interpreted with caution and viewed as exploratory. Nevertheless, by triangulating survey data with in-depth interviews in multiple institution types, our study provides a useful first step towards understanding how physicians perceive the clinical and nonclinical use of PGHD.

### Conclusions

Overall, the results of our survey reveal a consistent narrative: Physicians are cautiously optimistic about the value of PGHD, provided certain conditions are met. Across both large tertiary hospitals and smaller institutions, doctors emphasize the need for accurate, reliable data and better integration tools to make the use of PGHD feasible in everyday practice. The key differences by clinical setting were modest, mainly reflecting that tertiary centers have younger staff and somewhat better access to PGHD infrastructure (though not universally used), whereas smaller hospitals face more definite resource gaps (lack of systems, less exposure to PGHD). However, both groups converged on the view that system improvements (integration and standardization) and support (time, guidelines, and security) are essential. Physicians were primarily concerned with the accuracy of the data and the additional workflow burden, and prioritized PGHD uses that aligned with improving care, advancing research, or public health, rather than commercial interests. These insights suggest that efforts to implement PGHD should focus on building reliable systems that minimize burden, for example, by integrating PGHD into EHRs with automated summarization, providing clinical guidelines for interpretation, and compensating for additional workload. In parallel, policy and regulatory actions (such as setting data standards, improving privacy protections, and clarifying legal responsibilities) will be crucial to address physicians’ concerns and pave the way for the greater adoption of PGHD in both clinical practice and secondary applications.

Taken together, our findings suggest that PGHD can improve outpatient care and provide value to research and public health. However, its effective integration requires the following: standardized collection tools to ensure accuracy and comparability, visualization, and contextualization to reduce interpretive burden and facilitate patient–physician communication, and robust but flexible governance frameworks that address security, transparency, and consent. Future research should evaluate not only technical integration strategies but also mechanisms to ensure equitable participation across specialties and patient populations. Ultimately, the sustainable adoption of PGHD will depend on the balance of its potential benefits against the realities of clinical workflow and the trust of both physicians and patients.

## Data Availability

The data pertaining to this study’s results are available from the Asan Medical Center Institutional Data Access/Ethics Committee. Public disclosure of the data is not feasible because data sharing was not planned during study design and Institutional Review Board review. Researchers who meet the criteria for access to confidential data may contact the committee at irb@amc.seoul.kr or +82-2-3010-7166.

## Abbreviations

EHR: electronic health record
GDPR: General Data Protection Regulation
PGHD: patient-generated health data
PHR: patient health record
